# Oat Beta-Glucan Dietary Intervention on Antioxidant Defense Parameters, Inflammatory Response and Angiotensin Signaling in the Testes of Rats with TNBS-Induced Colitis

**DOI:** 10.3390/nu16152546

**Published:** 2024-08-03

**Authors:** Michał Oczkowski, Katarzyna Dziendzikowska, Anna Pasternak-Winiarska, Kuba Jarmołowicz, Joanna Gromadzka-Ostrowska

**Affiliations:** Department of Dietetics, Institute of Human Nutrition Sciences, Warsaw University of Life Sciences (SGGW), Nowoursynowska 166, 02-787 Warsaw, Poland; katarzyna_dziendzikowska@sggw.edu.pl (K.D.); a.pasternak.winiarska@gmail.com (A.P.-W.); kuba.jarmolowicz92@gmail.com (K.J.); joanna_gromadzka-ostrowska@sggw.edu.pl (J.G.-O.)

**Keywords:** colitis, inflammatory bowel disease (IBD), oat beta-glucan (OBG), oxidative stress, pro-inflammatory cytokines, renin–angiotensin system, testes

## Abstract

Male infertility represents a significant public health concern. There is a negative impact of inflammatory bowel diseases (IBDs) on the male reproductive system. The aim of this study was to investigate whether oat beta-glucan (OBG) with different molar mass can modulate parameters of antioxidant defense and inflammatory response in the testes of adult Sprague–Dawley rats with TNBS-induced colitis and whether the OBG intervention can modulate the inflammatory response in association with the RAS system. Results: higher testicular superoxide dismutase (SOD), glutathione reductase (GR) activities and glutathione (GSH) concentration, and lower testosterone (T) level and glutathione peroxidase (GPx) activity, were observed in rats with colitis than in healthy control ones. TNBS-induced colitis resulted in decreased the angiotensin 1–7 (ANG 1–7) level in the testes of rats fed with low-molar mass OBG compared to control animals. Conclusions: although colitis induced moderate pro-oxidant changes in the gonads, it seems plausible that dietary intervention with different fractions of oat beta-glucans mass may support the maintenance of reproductive homeostasis via the stimulation of the local antioxidant defense system.

## 1. Introduction

Infertility affects both women and men, but a notable increase in the prevalence of male infertility, estimated at 30–50%, is observed in men. In 2019, the estimated global prevalence of male infertility was more than 56 million cases [1]. The causes of declining fertility in men are multifactorial, with numerous factors influencing the outcome [2].

It is proposed that dysfunction of the male reproductive system may be also associated with chronic inflammation in the body, which may not originate in the reproductive system [3]. In this context, the presence of inflammatory bowel disease (IBD) is regarded as a potential contributing factor to male infertility [4].

IBD is a group of chronic inflammatory disorders of the gastrointestinal tract, primarily affecting the small intestine and colon. The most prevalent subtypes of IBD are Crohn’s disease and ulcerative colitis (UC), which exhibit differences in both their anatomic locations within the gastrointestinal tract (GIT), as well as in their underlying pathogenic processes. CD is defined by the presence of chronic inflammation in the entire thickness of the intestinal wall, and it can manifest in the other regions of the GIT. In contrast, UC is characterized by inflammation limited to the mucosa and submucosa of the colon. The chronic inflammatory state in IBD, driven by an overactive immune response, leads to elevated levels of pro-inflammatory cytokines (TNF-α, IL-1β and IL-6) in the blood [5].

Recent studies demonstrated a correlation between gut-mediated inflammation and the dysregulation of male reproductive system functions [6] leading to a higher risk of infertility among IBD patients than in the general population. Moreover, the results from human studies [7] have revealed lower sperm quality in men with IBD compared with healthy control subjects. Similar findings were reported in the study by Darmadi et al. [8], which demonstrated a negative correlation between the severity of IBD symptoms and semen parameters and serum testosterone (T) level. As IBD severity increased, there was a corresponding decline in serum T concentration, sperm concentration, motility and morphology. There are several possible explanations for this association. The injury in the small intestine and colon present in IBD conditions activates enzymes (such as myeloperoxidase) to produce reactive oxygen species (ROS) in the tissue. Secondly, the chronic inflammation noted in IBD patients contributes to the formation of pro-inflammatory cytokines in the testes [9]. Additionally, the therapeutic drugs against IBD symptoms reveal anti-fertility effects. The results of studies using animal models with DSS- or TNBS-induced IBD revealed excessive oxidative stress, impaired spermatogenesis and steroidogenesis in the gonads, which is supported by an increased level of pro-inflammatory cytokines that migrate from the damaged intestine, via the systemic circulation, to the testes. The diminished intestinal barrier plays a pivotal role in the stimulation of systemic inflammation that affects testicular function [10].

Male gonadal functions are regulated by a complex interplay of hormones, including gonadotropins, androgens and various paracrine and autocrine factors that facilitate testicular homeostasis. In this context, the endocrine regulator, the renin–angiotensin system (RAS), with its pleiotropic actions, is also involved in the modulation of immune response and organ damage [11], including in the male gonads [12].

Plant-derived compounds with antioxidant activity are useful in the treatment of testicular dysfunction [13]. Oat beta-glucan (OBG), a non-starch oat polysaccharide, has been shown to have beneficial health effects in a number of studies [14,15]. The findings of our previous research indicated that an OBG dietary intervention has the potential to exert immunomodulatory and anti-inflammatory effects in an IBD animal model [16,17].

The existing data on the impact of beta-glucans on the modulation of the male reproductive system are limited. Furthermore, the available data suggest that beta-glucans derived from different sources may enhance semen quality and reduce oxidative stress [18,19].

Considering that IBD is associated with gonadal dysfunction and plant bioactive compounds exert antioxidant and anti-inflammatory effects in the intestine and gonads, it is important to design an appropriate dietary intervention, which may be a simple solution to improve the functioning of the mentioned organs. In our in vivo model, 2,4,6-trinitrobenzenesulfonic acid (TNBS) induced colitis in rats, causing changes mainly characteristic of Crohn’s disease, including transmural colitis [20]. To the best of our knowledge, there are no data describing the potential role of OBG dietary intervention in preventing IBD-induced reproductive disruption. Therefore, the aim of the present study was to investigate whether OBG with different molar mass added to animal feed could modulate parameters of antioxidant defense and inflammatory response in the testes of rats with TNBS-induced colitis. An additional aim of this study was to investigate whether OBG intervention in IBD animals could modulate the inflammatory response in association with the RAS system.

## 2. Material and Methods

### 2.1. Oat Beta-Glucan (OBG) Preparation and In Vivo Experiment

The OBG isolates of high and low molar masses were prepared from the oat bran by the patented method described in previous work [21,22]. Briefly, the alkaline extraction and subsequent precipitation at the isoelectric point were used to remove the remaining proteins in the OBG isolate. Next, the obtained preparation was purified with proteolytic, pectinolytic and amylolytic enzymes. To maintain the final fraction of OBG in the range of nominal molecular mass, the conditions of the process were appropriately selected. The molar mass was determined using HPLC-SEC according to commercially available standards of (1-3)(1-4)-beta-d-glucan. The adult male outbred Sprague–Dawley Crl:CD(SD) rats (initial weight 415.0 ± 1.5 g, n = 38) were purchased from The Charles River Laboratories (Sulzfeld, Germany). This Sprague–Dawley rat strain is commonly used as an animal model for colitis. All animals were kept in the animal house of the Institute of Veterinary Medicine at Warsaw University of Life Sciences, Poland under standard conditions (temperature: 22 ± 1 °C, relative humidity: 50 ± 5% relative, light/dark cycle: 12/12 h). The rats were housed in individual polyurethane cages with access to water and AIN-93M feed ad libitum. To enrich the environment in rats cages we used glass balls and metal round items. All experimental procedures were approved by the 2nd Local Animal Care and Use Committee, Warsaw, Poland (Resolution No. 60/2015) and were in accordance with the UE Directive (2010/63/UE), the Polish legal regulation, and with respect to the 3R (Replacement, Reduction and Refinement) rules.

### 2.2. Induction of Colitis and Treatment Procedures in Animal Experiment

**Figure 1:**
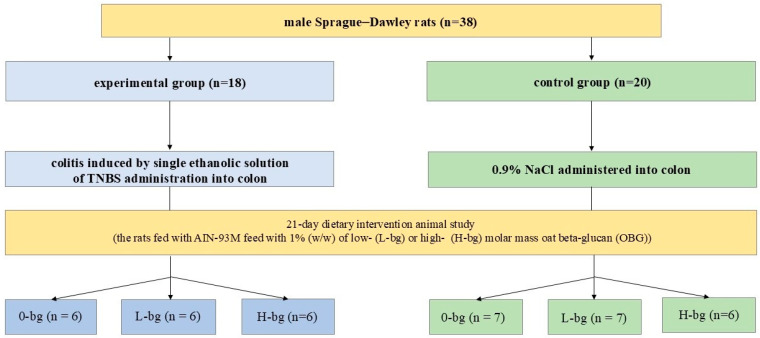
The scheme of experimental design of the study. TNBS, 2,4,6-trinitrobenzenesulfonic acid.

After one week of acclimatization, the animals were assigned to two main groups (experimental and control). Animals from the experimental group (n = 18) were subjected to an ethanolic solution of 2,4,6-trinitrobenzenosulfonic acid (TNBS, 150 mg/kg of rats body weight, Sigma-Aldrich, Darmstadt, Germany) by single intracolonic administration using a polyethylene flexible catheter (TP-18-75-50; Instech Laboratories, Inc., Plymouth Meeting, PA, USA) to induce acute colon inflammation (colitis). Simultaneously, the animals from the control group (n = 20) were subjected to 0.9% NaCl in the same way. The procedure was performed in animals under light Isoflurane (Aerrane Isoflurane USP, Baxter S.A, Lessines, Belgium) anesthesia. After TNBS/saline administration, the animals from the experimental and control group were divided into three dietary subgroups (Figure 1), depending on the molar mass of OBG: (1) rats received AIN-93M feed with 1% (*w*/*w*) of low molar mass (5.9 × 10^4^ ± 0.3 × 10^4^ g/mol) OBG (L-bg groups), (2) rats received AIN-93M feed with 1% (*w*/*w*) of high molar mass (1.7 × 10^6^ ± 0.05 × 10^6^ g/mol) OBG (H-bg groups) and (3) animals fed with AIN-93M feed without OBG (0-bg groups). The number of animals in experimental groups was calculated in accordance with the methodology proposed by Dell et al. [23], utilizing the tools for estimating the sample size in in vivo experiments.

The physicochemical characteristics of OBG fractions were presented in a previously published paper [21]. The animals were maintained in the experiment for 21 days, during which their health status was monitored every day. Moreover, the feed intake and animal weights were recorded every second day and once a week, respectively. After the experiment, animals were anesthetized by Isoflurane (Aerrane Isoflurane USP, Baxter S.A, Lessines, Belgium) inhalation and bled up by heart puncture. During the experimental period, the animals did not reveal any symptoms indicating a disturbance in their well-being, therefore the humane endpoints were not used. Peripheral blood samples were collected in EDTA-coated tubes and blood plasma was obtained by centrifugation of blood at 2300× *g* for 10 min and stored at −80 °C until further testosterone analysis. The testes were dissected, frozen in liquid nitrogen and stored at −80 °C until further biochemical analysis.

### 2.3. Testosterone (T) Concentration in Plasma and Testes

Plasma and testicular T concentration determined using ELISA kit (#40251, Neogen Testosterone Elisa kit, Lansing, MI, USA; intra- and inter-assay precision: <10%, respectively; sensitivity: 80% B/B_0_ = 0.006 ng/mL, 50% B/B_0_ = 0.029 ng/mL, assay range: 0.002–0.2 ng/mL) according to the manufacturer’s instruction. The steroid extraction from tissue was performed according to the method described in detail in previously published work [24].

### 2.4. Antioxidant Defense Parameters

Total superoxide dismutase (SOD), glutathione peroxidase (GPx) and reductase (GR) activities in testes were determined spectrophotometrically using a commercially available assay kits purchased from Cayman Chemical Company, Ann Arbor, MI, USA (cat. no.: #706002, #703102, and #703202 for SOD, GPx and GR, respectively), and were performed according to the manufacturer’s instructions. The reduced glutathione (GSH) and glutathione disulfide (GSSG) concentrations in testes were analyzed spectrophotometrically using an assay kit (cat. No #703002) purchased from Cayman Chemical Company, Ann Arbor, MI, USA. The concentration of reduced GSH in testes was calculated from the difference between the assayed total GSH and GSSG. The samples were analyzed in duplicate.

### 2.5. Pro-Inflammatory Cytokines Level in Testes

The level of selected pro-inflammatory cytokines in testes was analyzed using rat-specific ELISA kits (IL-1β: #ER1094, Fine Biotech Co., Wuhan, China; intra- and inter-assay precision: <8% and <10%; sensitivity <18.75 pg/mL, detection range: 31.25–2000 pg/mL; TNFα: #E0079r, EIAab Science, Wuhan, China; intra- and inter-assay precision: ≤6.9% and ≤10.9%; detection range: 15.6–1000 pg/mL; IL-6: #E0133r, EIAab Science, Wuhan China; intra- and inter-assay precision: ≤4.5% and ≤6.9%; detection range: 15.6–1000 pg/mL). Tissue homogenates were prepared according to the manufacturer’s instructions. The samples were analyzed in duplicates and adjusted to the protein content that was measured using Pierce™ BCA Protein Assay kit (ThermoScientific, Rockford, IL, USA) according to manufacturer’s instructions, using a microplate reader (Anthos Zenyth 200rt, Biochrom Ltd., Cambridge, UK).

### 2.6. RAS System Components Protein Level in Testes

The level of angiotensin II (ANG II), angiotensin III (ANG III) and angiotensin 1–7 (ANG 1–7), as well as receptors ATR1 (for ANG II) and MasR (for ANG 1–7), in testes were determined using rat-specific ELISA kits (ANG II: #201-11-1753, SunRedBio, Shanghai, China; intra- and inter-assay precision: <10% and <12%, respectively; sensitivity: 5773 pg/mL, assay range: 6–1800 pg/mL; ANG III: #201-11-1718, SunRedBio, Shanghai, China; intra- and inter-assay precision: <8% and <11%, respectively, sensitivity: 1.858 pg/mL, assay range: 2–600 pg/mL; ANG 1–7: #CES085Ra, Cloud-Clone Corp., Wuhan, China; intra- and inter-assay precision: <10% and <12%, respectively, sensitivity: 3.95 pg/mL, detection range: 9.88–800 pg/mL; ATR1: #SEB658Ra, Cloud-Clone Corp., Wuhan, China; intra- and inter-assay precision: <10% and <12%, sensitivity: 0.059 ng/mL, detection range: 0.156–10 ng/mL; MasR: #SEH683Ra, Cloud-Clone Corp., Wuhan, China; intra- and inter-assay precision: <10% and <12%, respectively; sensitivity: 0.059 ng/mL, detection range: 0.156–10 ng/mL). Tissue homogenates were prepared in accordance with the manufacturer’s instructions. The samples were analyzed in duplicate and adjusted to the protein content according to the previously described method.

### 2.7. Statistical Analysis

Statistical analyses were executed with Statistica software version 13.3 (TIBCO Software Inc., Palo Alto, CA, USA). The results were presented as mean ± SEM. Differences between groups were investigated using a two-way analysis of variance (ANOVA) with Duncan’s post hoc test. Differences were considered as significant at *p* < 0.05. Fisher’s linear discriminant analysis (F-LDA) was performed using R statistical software v. 3.3.3. (www.rproject.org/ (accessed on 3 October 2023)) (R: The R Project for Statistical Computing). This analysis was utilized to evaluate the interaction between the investigated parameters, aiming to identify linear combinations of these parameters that best separate predefined groups.

## 3. Results

### 3.1. Feed Intake and Body Weight Gains

The rats’ feed intake and weight gains depended on the animals’ health condition, with lower feed intake and lower weight gains in animals with TNBS-induced colitis compared to control animals. The post hoc analysis confirmed that the animals with colitis from the H-bg and 0-bg groups had lower food intake and weight gains compared to those from the L-bg group (with colitis) and relevant control animals, respectively (Table 1).

### 3.2. Plasma and Testicular Testosterone Concentration

**Figure 2:**
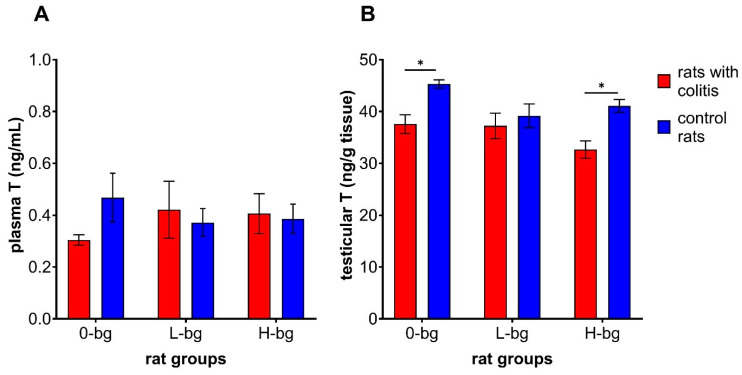
Testosterone concentration in plasma (**A**) and in testis (**B**) of rats with TNBS-induced colitis; 0-bg, rats fed with control feed (without OBG); L-bg, rats fed with feed supplemented with 1% of low-molar mass OBG; H-bg, rats fed with feed supplemented with 1% of high molar mass OBG. Data are expressed as mean ± SEM; *—denotes a statistically significant difference at *p* < 0.05; Two-way analysis of variance (ANOVA) with Duncan’s post hoc test.

Analyzing the changes in plasma T concentration (Figure 2A), it was observed a large individual variation in results between rats from experimental or control groups. However, no significant changes were observed in plasma T concentration depending on the experimental factors (ANOVA, NS) were observed. As for testicular T concentrations (Figure 2B), rats with TNBS-induced colitis revealed lower T concentrations than control animals (ANOVA, *p* < 0.001). Additionally, the animals fed with a diet supplemented with OBG exhibited lower T in testes compared to animals maintained on control feed (ANOVA, *p* < 0.05). In particular, the post hoc analysis showed a lower T concentration in the testes of IBD rats fed with control feed or high molar mass OBG.

### 3.3. Antioxidant Defense Parameters in Testes

**Figure 3:**
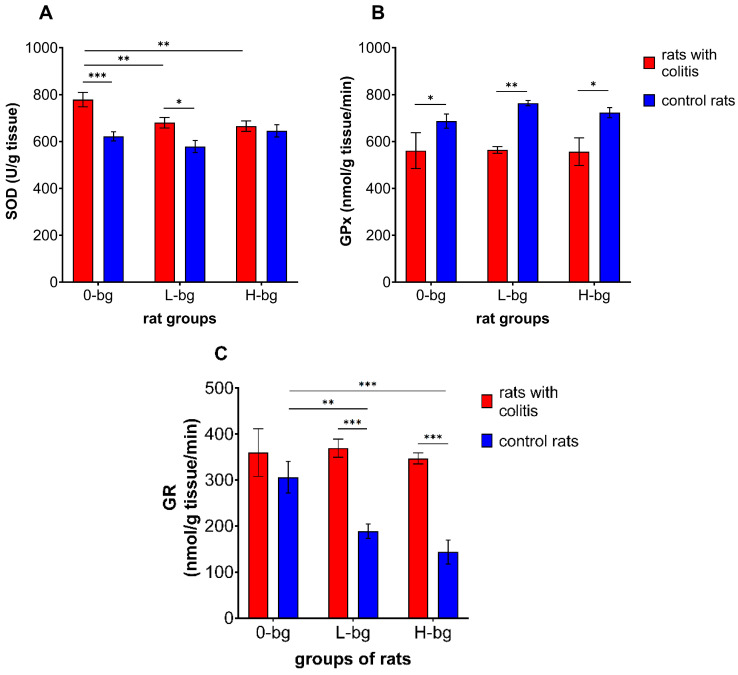
Activity of superoxide dismutase (SOD) (**A**), glutathione peroxidase (GPx) (**B**) and glutathione reductase (GR) (**C**) in testes of rats with TNBS-induced colitis; 0-bg, rats fed with standard feed (without OBG); L-bg, rats fed with feed containing 1% of low molar mass OBG; H-bg, rats fed with feed containing 1% of high molar mass OBG. Data are expressed as mean ± SEM; *^,^**^,^***—denote statistically significant differences at *p* < 0.05, *p* < 0.01 and *p* < 0.001, respectively; two-way analysis of variance (ANOVA) with Duncan’s post hoc test.

The effects of dietary intervention with OBG on antioxidative defense enzymes in rats with TNBS-induced colitis are shown in Figure 3A–D. The colon inflammation elicited the increased activity of SOD (ANOVA, *p* < 0.0001) (Figure 3A). Analyzing the impact of experimental factors, the statistical analysis revealed that SOD activity was significantly related to dietary supplementation (*p* < 0.05) and colitis (*p* < 0.0001). Higher enzyme activity was observed in animals with colitis compared to healthy ones. We observed that feeding the animals with dietary OBG resulted in decreased SOD activity. The post hoc analysis showed significantly higher SOD activity in animals with colitis fed with control feed or fed supplemented with low-molar mass OBG. The two-way ANOVA results confirmed the GPx activity in testes was significantly lower in rats with TNBD-induced colitis compared with the control animals (ANOVA, *p* < 0.001), irrespective of dietary intervention (ANOVA, NS) (Figure 3C).

On the other hand, GR activity in testes significantly differed depending on both experimental factors (ANOVA: colitis, *p* < 0.001 and dietary intervention, *p* < 0.05). As presented in Figure 3C, the animals with colitis revealed higher GR activity compared to the control animals. It was also observed that dietary intervention with low or high molar mass OBG resulted in diminished GR activity compared to the control group. Furthermore, it was also observed that a diet containing OBG resulted in stronger inhibition of testicular GR activity in control animals compared with rats with colitis.

**Figure 4:**
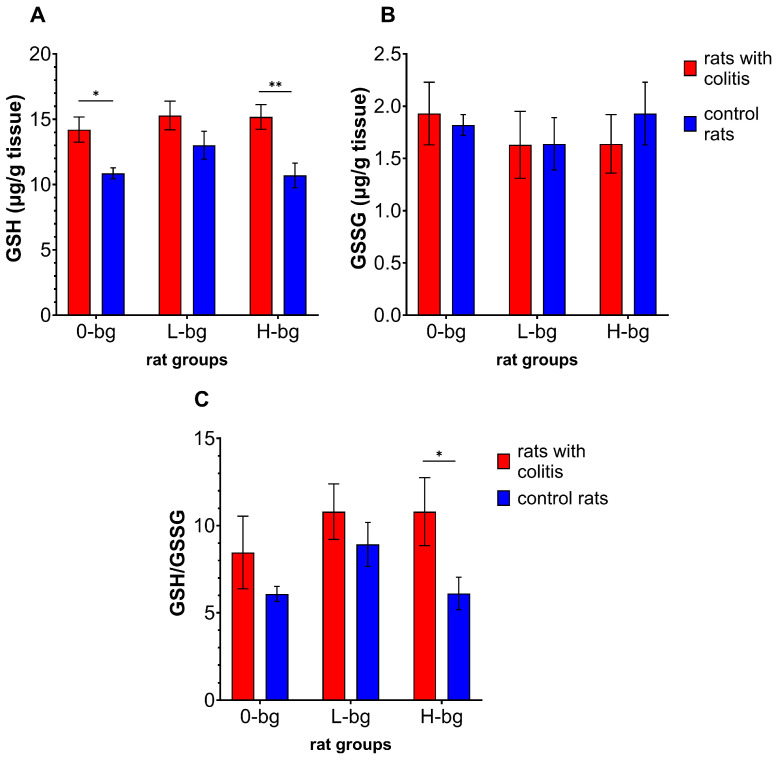
Testicular concentration of glutathione (GSH) (**A**), glutathione disulfide (GSSG) (**B**) and GSH to GSSG ratio (**C**) in rats with TNBS-induced colitis; 0-bg, rats fed with standard feed (without OBG); L-bg, rats fed with feed containing 1% of low molar mass OBG; H-bg, rats fed with feed containing 1% of high molar mass OBG. Data are expressed as mean ± SEM; *^,^ **—denotes a statistically significant difference at *p* < 0.05 and *p* < 0.01, respectively; two-way analysis of variance (ANOVA) with Duncan’s post hoc test.

Figure 4A–C presents the results of two forms of glutathione (GSH and GSSG) levels, and the GSH to GSSG ratio in the testes. Induction of colitis in ratsresulted in significant changes in the level of GSH and the ratio of GSH to GSSG between experimental groups (ANOVA, *p* < 0.01). The statistical analysis revealed that GSH level was significantly higher in IBD rats fed with the control diet or with high molar-mass OBG-containing feed compared to healthy rats. Although we did not observe significant changes in GSSG level depending on the experimental factors (ANOVA, NS), the ratio of GSH to GSSG was dependent on colon inflammation (ANOVA, *p* < 0.05). The post hoc analysis demonstrated a significantly higher ratio of the two forms of glutathione in IBD animals fed with high-molar mass OBG than in control animals.

### 3.4. Pro-Inflammatory Cytokines Level in Testes

**Figure 5:**
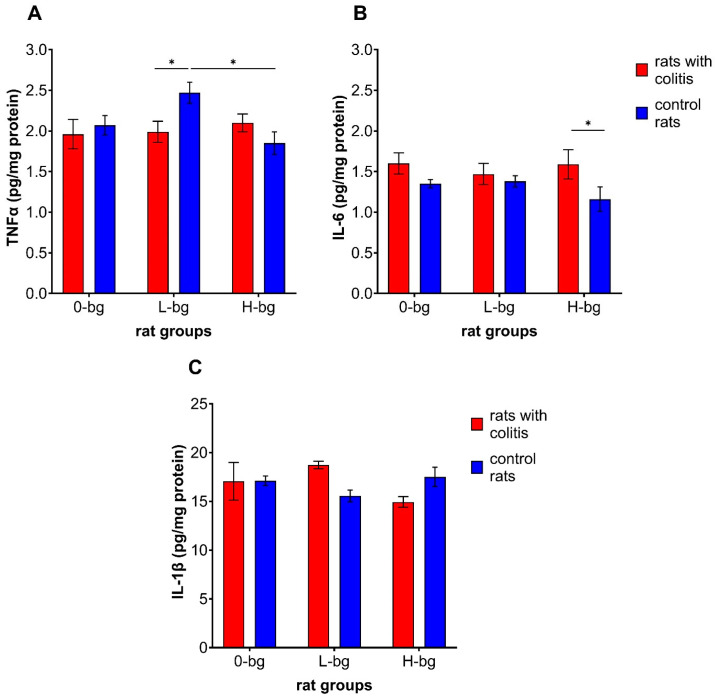
Testicular concentration of TNF-α (**A**), interleukin 6 (IL-6) (**B**) and interleukin 1β (IL-1β) (**C**) in rats with TNBS-induced colitis; 0-bg, rats fed with standard feed (without OBG); L-bg, rats fed with feed containing 1% of low molar mass OBG; H-bg, rats fed with feed containing 1% of high molar mass OBG. Data are expressed as mean ± SEM; *—denotes statistically significant difference, * *p* < 0.05; two-way analysis of variance (ANOVA) with Duncan’s post hoc test.

As illustrated in Figure 5A, neither TNBS-induced colitis nor dietary intervention had a significant impact on the level of TNFα in the testis (ANOVA, NS). However the interaction between experimental factors revealed a significantly higher cytokine level in the testis of control rats receiving a diet containing low molar mass OBG compared with the animals with colitis fed the same type of diet and with animals fed with a diet with a high molar mass OBG. With regard to IL-6 level in the testes, our findings revealed a notable impact of colitis, as evidenced by the ANOVA results (*p* < 0.01). Additionally, we observed a higher IL-6 level in the testes in animals exposed to colon inflammation. The post hoc analysis has shown that, in the case of rats fed with feed supplemented with high molar mass OBG, the significantly higher IL-6 level in the testes was observed in rats exposed to colon inflammation compared control rats (Figure 5B). At the same time, no significant changes in IL-1β in the testes of animals between experimental and control groups were observed (Figure 5C).

### 3.5. Renin–Angiotensin System Compound Level in the Testes

**Figure 6:**
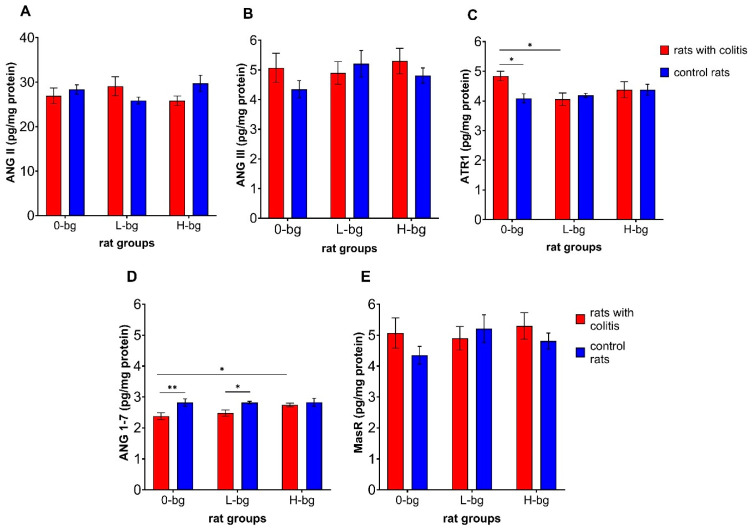
Angiotensin II (ANG II) (**A**), angiotensin III (ANG III) (**B**), receptor for angiotensin II (ATR1) (**C**), angiotensin 1–7 (ANG 1–7) (**D**) and MasR receptor for ANG 1–7 (**E**) protein level in testes of rats with TNBS-induced colitis; 0-bg, rats fed with standard feed (without OBG); L-bg, rats fed with feed containing 1% of low molar mass OBG; H-bg, rats fed with feed containing 1% of high molar mass OBG. Data are expressed as mean ± SEM; *—denotes statistically significant difference, * *p* < 0.05; ** *p* < 0.01, two-way analysis of variance (ANOVA) with Duncan’s post-hoc test.

The analysis of RAS parameters in the testes revealed a significant interaction (*p* < 0.05) between experimental factors on the ATR1 level (Figure 6C) and a significant impact of IBD (*p* < 0.01) on the level of ANG 1–7 (Figure 6D). Simultaneously, we did not observe a significant impact of experimental factors in relation to ANG II (Figure 6A), ANG III (Figure 6B) and MasR (Figure 6E). The level of ATR1 was significantly higher in IBD animals fed with a control diet compared with their healthy counterparts and compared to IBD rats receiving a diet with low-molar mass OBG (Figure 6C). In turn, the level of ANG 1–7 in IBD rats fed with control feed was significantly lower than in healthy rats without OBG dietary intervention and compared with IBD animals fed with feed containing high-molar mass OBG.

Fisher’s linear discriminant analysis (FLD) was conducted to investigate the differences in the parameters associated with testis functions and the effects of OBG supplementation in animals with IBD. This analysis was employed to derive the linear combination of antioxidative defense markers, oxidative stress parameters and components of the RAS in the testes, facilitating the optimal separation of the experimental groups. The FLD method enabled the identification of the most discriminative features, providing a robust statistical model for classification and enhancing our understanding of the intricate relationships within the dataset.

**Figure 7:**
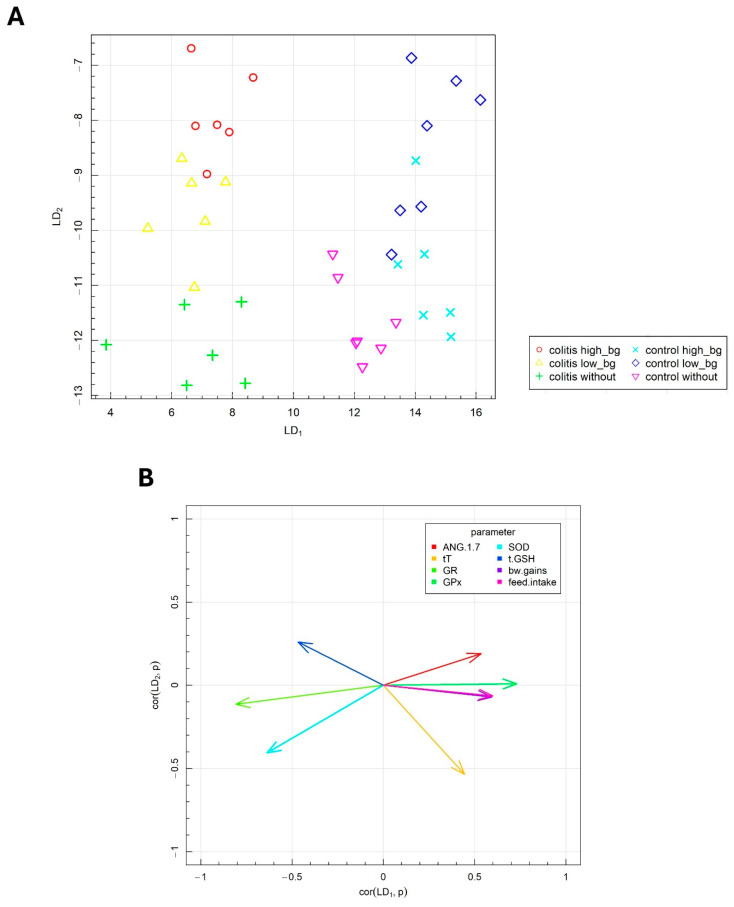
Fisher’s linear discriminant analysis (F-LDA): (**A**) experimental by two the most data separating FLDs and (**B**) parameters contributing the most to FLDs.

The results of Fisher’s Linear Discriminant Analysis (F-LDA) for the experimental data are presented in Figure 7A,B. In Figure 7A, the six treatment groups were distinctly separated. The data are displayed in the space defined by the linear combinations of parameters (FLDs), labeled as FLD1 and FLD2, which ensure optimal separation of the predefined groups. Figure 7B depicts the correlation vectors of the studied parameters with the FLD1 and FLD2 predictors, illustrating the direction and magnitude of each parameter’s influence on the separation of the experimental groups.

The biological markers corresponding to specific vectors caused the data to shift in the direction indicated by these vectors. As indicated in the horizontal plane (FLD1), the animal groups with colitis were distinctly separated from the healthy animals. The F-LDA confirmed the findings from the ANOVA, which demonstrated the significant effects of TNBS-induced colitis on the investigated parameters, notably, the reduced body weight gain, decreased food intake and lower T concentration in testes of animals with colitis. Additionally, the separation between colitis and healthy animals was facilitated by the changes in antioxidant defense parameters, specifically, the decreased GPx activity and the increased activities of SOD, GR and GSH levels in the testes of rats with colitis.

The FLD analysis also provided insights into the combination of parameters that distinguish the effects of different molar masses of OBG supplementation in the vertical plane (LD2). The vertical plane (LD2) facilitated the separation of the control group from the groups whose diets were supplemented with either low- or high-molar mass OBG. The effects of OBG varied slightly between healthy animals and those with colitis. The parameters that primarily differentiated the groups in response to oat OBG supplementation included SOD activity, GSH, T and ANG 1–7 level in testes.

## 4. Discussion

Extensive research has been conducted to ascertain the health benefits of oat beta-glucan, a polysaccharide with anti-inflammatory and antioxidant properties. These benefits have been demonstrated in particular in relation to chronic inflammatory bowel diseases [25] or for their hypolipidemic properties [26]. The results of the presented study indicate that this polysaccharide may also exert influence beyond the digestive system, including effects on redox balance and RAS parameters in the gonads.

Our results indicate that colon inflammation led to decrease in animal feed intake and weight gains. This observation was in accordance with the findings reported by Crespo et al. [27] in a Balb/c mouse model of TNBS-induced colitis. The induction of colon inflammation, accompanied by intestinal bleeding, diarrhea and pain, appears to be a significant stressor for animals that leads to decreased appetite [28].

It is established that IBD can induce alterations in the sex steroid hormone secretion. For instance, the diagnosis of IBD prior to puberty can result in a delay in sexual maturation and is independent of the other key factors (e.g., body weight and serum leptin level) regulating this process [29]. On the other hand, androgen therapy in men with IBD can reduce systemic inflammation, contributing to decreased production of pro-inflammatory cytokines (TNFα, IL-6 and IL-1β) [30]. The role of IBD in androgen metabolism and their peripheral blood levels are a relatively understudied area. However, the recently discovered link between disruption of the microbiota and steroid hormone metabolism has provided new insights. Interesting findings were proposed by Colldén et al. [31], who demonstrated that the gut microbiota is involved in androgen metabolism, regulating their peripheral levels. Findings from human studies [8] have indicated that male patients experiencing severe IBD symptoms demonstrated the lowest serum-free and total testosterone concentrations compared with individuals experiencing mild disease symptoms. Although our study did not reveal any significant differences in plasma T levels, it can be suggested that colon inflammation may disrupt the microbiota profile, causing alterations in peripheral T levels. Alternatively, the lack of significant fluctuations in plasma T levels in animals with experimentally-induced colitis, as evidenced in our study, may also be due to the absence of substantial alterations in pro-inflammatory cytokine levels. The decreased gonadal T level found in our rats with colitis may be related to increased inflammatory markers, such as IL-6. It has been demonstrated that colitis has disrupted colon epithelial integrity and transferred bacterial endotoxins into the peripheral circulation to stimulate the immune cells to produce pro-inflammatory cytokines [32]. As was found by the other authors, during LPS-induced endotoxemia, testicular macrophages are stimulated to produce reactive oxygen species (ROS), which, in turn, leads to impairment of Leydig cells’ mitochondria and leads to decreased steroidogenesis [33,34]. As observed in our study, changes in testicular T concentration in rats with IBD may indicate a moderate stimulating effect of the low-molar mass OBG on steroidogenesis in animals with IBD.

Given that oxidative stress represents a prevalent mechanism linked to inflammatory processes within cells and tissues, our research also entailed an evaluation of the activity of pivotal enzymes (SOD, GPx and GR) involved in redox balance within the gonads. The observed alterations in the enzymes associated with redox balance in our study suggest a compensatory response in the gonadal antioxidative defense system rather than an adaptive response to increased ROS production following TNBS-induced colitis.

The findings from our study indicate that IBD affects the antioxidative defense system enzymes in gonads. On the other hand, the dietary intervention using two OBG fractions contributed to the changes in SOD and GR activities. The observations of GSH levels and the GSH to GSSG ratio demonstrated that the antioxidant defense was rather stimulated, particularly in animals with IBD that received feed with high-molar mass OBG. It is possible that this ‘beneficial’ effect was related to the compensatory stimulation of glutathione synthesis in the gonads of rats subjected to a single dose of TNBS and depended not only on the action of this OBG fraction itself but also on the time since the induction of IBD. In the case of animals that have been repeatedly exposed to DSS in drinking water, a weakening of antioxidant defense parameters was observed by Farombi et al. [35] and Chandra et al. [36]

Glutathione reductase (GR) is an enzyme that catalyzes the reduction of GSSG to reduced (GSH), which plays a crucial role in cellular antioxidant defense mechanisms [37]. In the current study, the rats with IBD exhibited higher GR activity in testes compared to control animals. However, OBG administration unexpectedly contributed to a reduction in GR activity without changes in GSSG and total testicular GSH levels. It can be postulated that the presence of inflammation in the colon may stimulate systemic mechanisms related to antioxidant defense, which in turn may increase the protection of the male gonads, as privileged organs due to their gametogenic function. The results of our previous study [38] indicated no alteration in GR activity in the peripheral blood of rats with colitis and dietary intervention with OBG. The observed reduction in GR activity in animals fed with OBG-containing feed may be associated with a relatively low testicular GSSG level. The findings from our study suggest that colon inflammation in rats resulted in decreased GPx activity irrespective of feed supplemented with OBG. This glutathione-dependent enzyme is responsible for the reduction of hydrogen peroxide or organic hydroperoxide molecules to water, thereby maintaining the cellular redox balance [39]. Our results demonstrated that colon inflammation resulted in lower GPx activity in the testes, but there were no effects of OBG supplementation. Our findings align with those previously reported by Farombi et al. [35]. A reduction in GPx activity may be attributed to the detoxification of free radicals generated within the gonads. With regard to the total GSH, our study revealed an increase in its level in the testes of animals with colon inflammation. This unexpected observation may be attributed to the stimulation of local, gonadal production of glutathione to improve the antioxidative defense and spermatogenesis in response to the excessive oxidative stress in the colon [40].

OBG is a molecule that has demonstrated beneficial effects in the gastrointestinal tract following the induction of inflammation [17]. The results of our study showed that IBD and dietary intervention had no impact on investigated pro-inflammatory cytokines levels in testes. Only the TNFα level in the control group of animals subjected to feed containing low-molar mass OBG was higher than in the relevant animal group with colitis. This effect could be mediated via the activation of toll-like (TLRs) and Dectin-1 receptors that are involved in the activation of the immune response [41,42] and can be considered as an immunomodulatory effect. In contrast to the results obtained in the current study, Parmar et al. [10] demonstrated that mice with DSS-induced colitis exhibited increased inflammation and cell damage in the testes.

We also investigated the impact of OBG dietary intervention on the RAS system components that are involved in the regulation of steroidogenesis and spermatogenesis [43]. Shibata et al. [44] have revealed that mice lacking the angiotensin-converting enzyme (tACE) exhibited reduced ATP production in male gametes. As demonstrated by Wang et al. [45], C57BL/6J wild-type mice treated with ANG II exhibited increased levels of 4-hydroxynonenal (4-HNE), an oxidative stress marker, and increased IL-6 expression in the testes. In contrast, the other authors have demonstrated that upregulation of RAS may be associated with pathological changes in the testes, as was shown by Wang et al. [46]. The results of our study indicated that colitis resulted in increased ATR1 level in testes than was observed in control animals fed with standard feed. At the same time, the animals with colitis fed with control feed or feed containing low-molar mass OBG exhibited decreased ANG 1–7 protein levels in the testes. ANG 1–7 and Mas receptor are ANG II antagonists and exert anti-inflammatory effects mediated through the inhibition of NFκB transcription factor activity and downregulation of TNFα, IL-1β, MCP-1 and CXCL1 [45,47]. ANG 1–7 reduces the activity of intracellular signaling molecules (p38 MAPK, ERK1/2 and JNK kinases) which play an important role in intensifying the inflammatory response [48].

## 5. Conclusions

The results obtained in this study may contribute to a better understanding of the mechanisms through which IBD affects the regulation of the reproductive system. Although colitis induced moderate pro-oxidant changes in the gonads, it seems plausible that dietary intervention with oat beta-glucans may support the maintenance of reproductive homeostasis via the stimulation of the local antioxidant defense system.

## Figures and Tables

**Table 1:** Feed intake and body weight gains in rats.

	Rat Groups					
Parameters	Rats with Colitis			Control Rats		
	0-bg (n = 6)	L-bg (n = 6)	H-bg (n = 6)	0-bg (n = 7)	L-bg (n = 7)	H-bg (n = 6)
feed intake (g/week)	172.5 ± 4.0	187.6 ± 6.6 ^#,##^	166.3 ± 9.1	191.3 ± 3.4 ^#^	197.9 ± 3.7	201.1 ± 2.2 ^###^
rats weight gain (% of initial body weight)	113.9 ± 1.1	118.6 ± 1.3 ^#,^^	114.5 ± 2.1	120.6 ± 0.8 **	121.8 ± 1.1	123.4 ± 0.9 ^^^^^

0-bg, rats fed with feed without OBG; L-bg, rats fed with feed supplemented with 1% of low-molar mass OBG; H-bg, rats fed with feed supplemented with 1% of high-molar mass OBG; ^#^ significantly different from 0-bg animals with colitis at *p* < 0.05; ^##,###^ significantly different from H-bg animals with colitis at *p* <0.01 and *p* < 0.001, respectively; ** significantly different from 0-bg animals with colitis at *p* <0.01); ^^,^^^^ significantly different from H-bg animals with colitis at *p* < 0.05 and *p* < 0.001, respectively; Two-way analysis of variance (ANOVA) with Duncan’s post hoc test, at *p* < 0.05.

## Data Availability

The original contributions presented in the study are included in the article, further inquiries can be directed to the corresponding author.
